# Supplementary material for: Deriving the skyrmion Hall angle from skyrmion lattice dynamics
Source: Nat Commun. 2021 May 11;12:2723. doi: 10.1038/s41467-021-22857-y (PMC8113591; doi:10.1038/s41467-021-22857-y)
Supplement: Supplementary file 3 — Description of Additional Supplementary Files [file 41467_2021_22857_MOESM3_ESM.docx]

Supplementary Movie 1:

Skyrmion lattice reorientation in a thin lamella of FeGe in the presence of a shear force, induced by a magnetic field gradient, measured via resonant elastic x-ray scattering (REXS). The skyrmion lattice was given 15 min to relax in the absence of a perturbative magnetic field gradient, indicated by frames with the “Current off” label. Afterwards, a current was driven through a wire to generate an out-of-plane magnetic field gradient in the sample. This field gradient generates a shear force in the lattice, which forces the lattice to reorient along the direction of motion. The effect of this shear force was measured for a further 15 min and the resulting frames are indicated with the “Current on” label. The video is sped up by a factor of 128 compared to the measurement time.

Supplementary Movie 2:

An average over 15 videos taken exactly as in Supplementary Movie 1. In all cases, the skyrmion lattice reorients itself along the direction of motion, but no net rotation is clearly visible as this video is an average over both clockwise and counterclockwise rotations.
